# 728. Potential Reservoirs for Additional Carbapenemase Gene Spread: Detection of Carbapenemase Genes in non-*Pseudomonas aeruginosa* and non-*Acinetobacter baumannii* isolates

**DOI:** 10.1093/ofid/ofad500.789

**Published:** 2023-11-27

**Authors:** Sarah Sabour, Alyssa Kent, Allison C Brown

**Affiliations:** Centers for Disease Control and Prevention, Atlanta, Georgia; CDC, Atlanta, Georgia; Centers for Disease Control and Prevention, Atlanta, Georgia

## Abstract

**Background:**

Public Health Laboratories (PHL) across 50 U.S. states, several cities and Puerto Rico participate in CDC’s Antimicrobial Resistance Laboratory Network (AR Lab Network), to test clinical isolates of carbapenem-resistant *Pseudomonas aeruginosa* (CRPA) and *Acinetobacter baumannii* (CRAB) for the presence of targeted carbapenemase (CP) genes. PHLs have submitted reports of these targeted genes in CR non-*aeruginosa Pseudomonas* (CR-NAP) and non-*baumannii Acinetobacter* (CR-NBA) isolates. Here we describe the ad-hoc detection of targeted CP genes in CR-NAP and CR-NBA species tested by the Network.

**Methods:**

Clinical laboratories submitted CR isolates to PHLs for organism identification. Isolates were deemed suspected CR-NAP and CR-NAB when a PHL ruled out *aeruginosa* or *baumannii*, respectively, but their MALDI ToF MS database prevented further speciation. PHLs used real-time polymerase chain reaction or whole genome sequencing to detect the presence of targeted CP genes: *bla*_KPC,_*bla*_NDM,_*bla*_OXA-48-like,_*bla*_IMP,_ and *bla*_VIM_ in *Pseudomonas* and *Acinetobacter,* and *bla*_OXA-23-like,_*bla*_OXA-24/40-like,_ and *bla*_OXA-58-like_ in *Acinetobacter*.

**Results:**

During 2017*–*2021, the AR Lab Network tested 56,092 CR-*Pseudomonas* isolates, 71 of which were CR-NAP; *P. putida* (n=15; 21%), *P. fluorescens* (12; 17%) *P. otitidis* (5; 7%), *P. monteilii* (3; 4%), 1 (1%) each of *P. koreensis* and *P. mosselii*, and 34 (48%) other suspected CR-NAP. Three (4%) of the 71 CR-NAP isolates tested carried a targeted CP gene: 1 *P. fluorescens* with *bla*_IMP_, 1 *P. putida with bla*_VIM_, and 1 *P. Japonica* with *bla*_KPC_.

Of 13,624 CR-*Acinetobacter* isolates tested, 25 (< 1%) were CR-NBA; *A. pitti* (10; 40%), *A. radioresistens* (6; 24%), *A*. *variabilis* (2; 8%), 1 (4%) each of *A. berezinaiae, A. lwoffii,* and *A. ursingii,* and 4 (16%) other suspected CR-NBA. The *A*. *variabilis* isolates each harbored both *bla*_NDM_ and *bla*_OXA-58-like_ genes.

**Conclusion:**

Although CR-NAP and CR-NBA isolates are not solicited by the Network, some of these species are submitted for characterization. Our findings suggest these other species do harbor some targeted CP genes and may represent unmonitored reservoirs for CP gene spread in the U.S. An expansion of testing to include these species in the AR Lab Network may improve our ability to slow the spread of antimicrobial resistance.

**Disclosures:**

**All Authors**: No reported disclosures

